# Quantifying esophageal motion during free-breathing and breath-hold using fiducial markers in patients with early-stage esophageal cancer

**DOI:** 10.1371/journal.pone.0198844

**Published:** 2018-06-11

**Authors:** Yoshiko Doi, Yuji Murakami, Nobuki Imano, Yuki Takeuchi, Ippei Takahashi, Ikuno Nishibuchi, Tomoki Kimura, Yasushi Nagata

**Affiliations:** Department of Radiation Oncology, Graduate School of Biomedical Sciences, Hiroshima University, Hiroshima City, Japan; North Shore Long Island Jewish Health System, UNITED STATES

## Abstract

**Introduction:**

Cardiac toxicity after definitive chemoradiotherapy for esophageal cancer is a critical issue. To reduce irradiation doses to organs at risk, individual internal margins need to be identified and minimized. The purpose of this study was to quantify esophageal motion using fiducial makers based on four-dimensional computed tomography, and to evaluate the inter-CBCT session marker displacement using breath-hold.

**Materials and methods:**

Sixteen patients with early stage esophageal cancer, who received endoscopy-guided metallic marker placement for treatment planning, were included; there were 35 markers in total, with 9, 15, and 11 markers in the upper thoracic, middle thoracic, and lower thoracic/esophagogastric junction regions, respectively. We defined fiducial marker motion as motion of the centroidal point of the markers. Respiratory esophageal motion during free-breathing was defined as the amplitude of individual marker motion between the consecutive breathing and end-expiration phases, derived from four-dimensional computed tomography. The inter-CBCT session marker displacement using breath-hold was defined as the amplitudes of marker motion between the first and each cone beam computed tomography image. Marker motion was analyzed in the three regions (upper thoracic, middle thoracic, and lower thoracic/esophagogastric junction) and in three orthogonal directions (right-left; anterior-posterior; and superior-inferior).

**Results:**

Respiratory esophageal motion during free-breathing resulted in median absolute maximum amplitudes (interquartile range), in right-left, anterior-posterior, and superior-inferior directions, of 1.7 (1.4) mm, 2.0 (1.5) mm, and 3.6 (4.1) mm, respectively, in the upper thoracic region, 0.8 (1.1) mm, 1.4 (1.2) mm, and 4.8 (3.6) mm, respectively, in the middle thoracic region, and 1.8 (0.8) mm, 1.9 (2.0) mm, and 8.0 (4.5) mm, respectively, in the lower thoracic/esophagogastric region. The inter-CBCT session marker displacement using breath-hold resulted in median absolute maximum amplitudes (interquartile range), in right-left, anterior–posterior, and superior-inferior directions, of 1.3 (1.0) mm, 1.1 (0.7) mm, and 3.3 (1.8) mm, respectively, in the upper thoracic region, 0.7 (0.7) mm, 1.1 (0.4) mm, and 3.4 (1.4) mm, respectively, in the middle thoracic region, and 2.0 (0.8) mm, 2.6 (2.2) mm, and 3.5 (1.8) mm, respectively, in the lower thoracic/esophagogastric region.

**Conclusions:**

During free-breathing, esophageal motion in the superior-inferior direction in all sites was large, compared to the other directions, and amplitudes showed substantial inter-individual variability. The breath-hold technique is feasible for minimizing esophageal displacement during radiotherapy in patients with esophageal cancer.

## Introduction

Recently, the efficacy of concurrent chemoradiotherapy (CCRT) for esophageal cancer has been reported [1–4]. CCRT has achieved favorable locoregional control and survival rates in early-stage esophageal cancer (EC) [3,4]. Given the progress in endoscopic diagnostic techniques, such as iodine staining and magnifying endoscopy with narrow band imaging, the screening of early-stage EC has become widespread. This trend has improved overall survival rate after definitive CCRT for EC.

However, cardiac toxicity after CCRT for EC is now a critical issue. Overall incidence rates are reported to be as high as 9.3–20.8% [5–9]. In radiotherapy (RT) for EC, irradiation fields should consider esophageal motion, and appropriate internal margins need to be attached to the clinical target volume (CTV). Large irradiation fields may lead to increase in the volume of the heart exposed to high-dose irradiation. Therefore, it is important to measure the physiological motion of the esophagus during free-breathing, and to minimize this motion.

In this study, we investigated physiological esophageal motion during free-breathing using the motion of fiducial markers, evaluated via respiratory-gated four-dimensional computed tomography (4D-CT). We also examined fiducial marker displacement using cone beam computed tomography (CBCT), to investigate the inter-CBCT session marker displacement using breath-hold.

## Materials and methods

### Patient data

**Fig 1:**
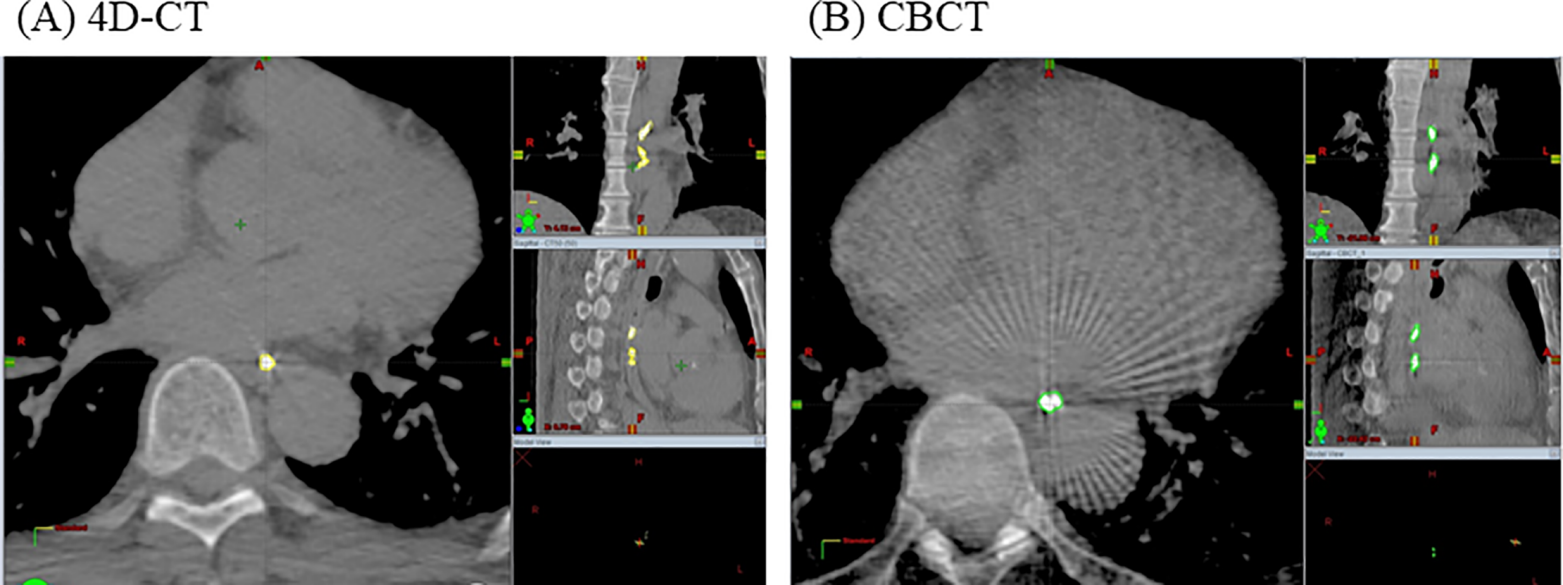
Contouring of metallic markers. Metal markers, placed by endoscopy to identify tumor location, were contoured in (A) four-dimensional computed tomography (4D-CT) and (B) cone beam CT (CBCT).

Sixteen patients with T1-2N0M0 esophageal cancer, according to the 7^th^ edition International Union Against Cancer Tumor-Node-Metastasis staging system [10], who received RT in our department, were included in this study (Table 1). All patients had squamous cell carcinoma histology. Three patients had simultaneous double primary lesions; thus, in total, we evaluated 19 tumors. The tumor depths were identified as mucosal (T1a), submucosal (T1b), and muscularis propria (T2) in 4, 10 and 5 cases, respectively. The tumor locations were identified as the upper thoracic esophagus (Ut), middle thoracic esophagus (Mt), and lower thoracic esophagus/esophagogastric junction (Lt) in 6, 7, and 6 cases, respectively. Metallic markers were placed at the cranial and caudal side of each tumor in all patients, for identifying tumor location and motion in RT treatment planning. In total, 35 markers were used, with 9, 15, and 11 placed in Ut, Mt, and Lt locations, respectively. These metallic markers were used as fiducial markers for esophageal motion, and all markers, identified on 4D-CT and CBCT images, were contoured by the same radiation oncologist (Y.D.) (Fig 1).

**Table 1:** Patient characteristics.

Gender	Male / Female	15 / 1
Age (years)	Median (range)	69 (59–82)
Tumor stage†	T1a / T1b / T2	4 / 10 / 5
Tumor location	Ut / Mt / Lt	6 / 7 / 6
Number of markers per patient	Average (range)	2 (1–4)
Location of markers	Ut / Mt / Lt	9 / 15 / 11

Abbreviations

† Three patients had simultaneous double primary tumors

Ut: upper thoracic esophagus; Mt: middle thoracic esophagus; Lt: lower thoracic esophagus/esophagogastric junction

All patients provided written informed consent for their clinical data to be included in the study analyses prior to treatment. The present study was reviewed and approved by the Institutional Review Board of Hiroshima University Hospital (approval number: E-881).

### Respiratory esophageal motion during free-breathing

We used respiratory-gated 4D-CT data to analyze respiratory esophageal motion during quiet free-breathing. 4D-CT scans were performed using the Varian Real-time Position Management (RPM) Respiratory Gating system (Varian Medical Systems, Palo Alto, CA). The CT data set was organized into 10-phase bins of the respiratory cycle, and phase-by-phase evaluation was performed on an AdvantageSim workstation (GE Healthcare, Princeton, NJ). The 10-phase sets ranged from 0% to 90% in steps of 10%, with either 0% or 90% as the 0% end-inspiratory phase and 50% as the end-expiratory phase. CT data sets were imported into the Eclipse radiotherapy treatment planning system (Varian Medical Systems, Palo Alto, CA); all markers were contoured and the centroidal point of each marker was calculated. In this study, we defined fiducial marker motion as motion of the centroidal point of the markers. The amplitude of individual marker motion, between the 10%-90% phase and 0% phase, was evaluated.

### The inter-CBCT session marker displacement using breath-hold

We investigated the inter-CBCT session marker displacement using CBCT images acquired after setting-up each patient’s position by bone-matching under breath-hold. Daily bone-matching was performed using orthogonal kV images, and digitally reconstructed radiographs (DRRs) were created from CT plans. The On-Board Imager system (Varian Medical Systems, Palo Alto, CA) was used to acquire kV images. Thoracic vertebral bodies on kV images were fused with those on DRRs images to determine shift values along the right-left (RL) / anterior-posterior (AP) / superior-inferior (SI) directions for bone-matching. We acquired kV-CBCT images using a full-scan method, which creates an image by rotating 360°. CBCT slice thicknesses were 2.5 mm. CBCT images were acquired 2 or 3 days from the beginning of treatment, and weekly thereafter. All CBCT data was imported into the Eclipse treatment planning system, and all markers were contoured. The inter-CBCT session marker displacement using breath-hold, between the first and each subsequent CBCT image, was evaluated.

### Data analysis

Marker motion was measured in 3D vector distances, as well as in RL (x-axis), AP (y-axis), and SI (z-axis) directions; positive values were indicative of right, anterior, and superior directions, respectively. We applied box-and-whisker plotting and the Wilcoxon signed-rank test to compare marker motion according to tumor site and motion direction. Probability values (P) < .05 were considered statistically significant. We also calculated the 95th percentiles values of absolute marker motion, generated from histograms and cumulative distribution curves.

## Results

### Respiratory esophageal motion during free-breathing

**Fig 2:**
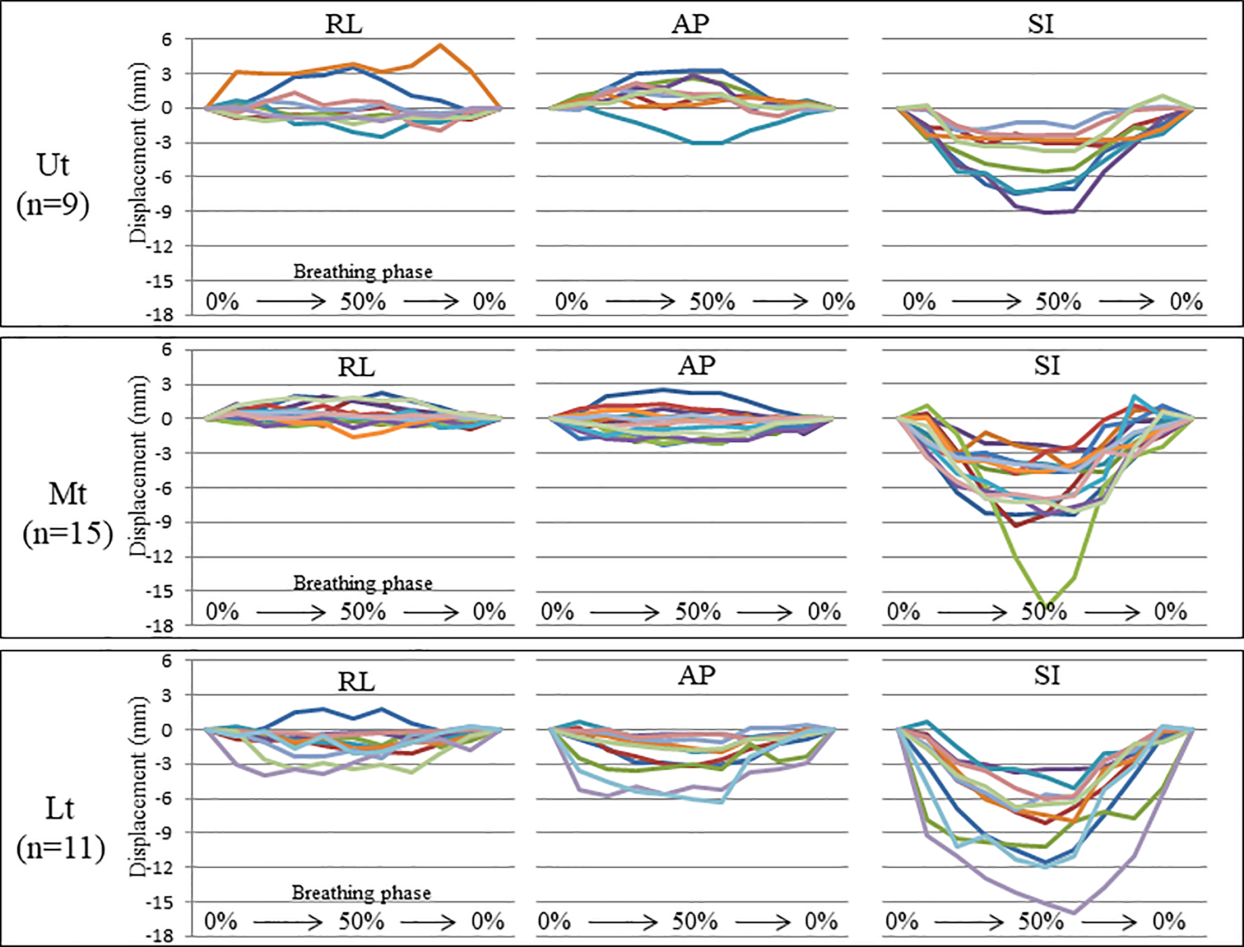
Plots of respiratory marker motion in consecutive breathing phases according to tumor site and motion direction. Plots of respiratory marker motion in consecutive breathing phases according to tumor site and motion direction. The different colors stand for the data from the different metal markers. Ut: upper thoracic esophagus; Mt: middle thoracic esophagus; Lt: lower thoracic esophagus /esophagogastric junction; RL: right–left; AP: anterior–posterior; SI: superior-inferior.

**Fig 3:**
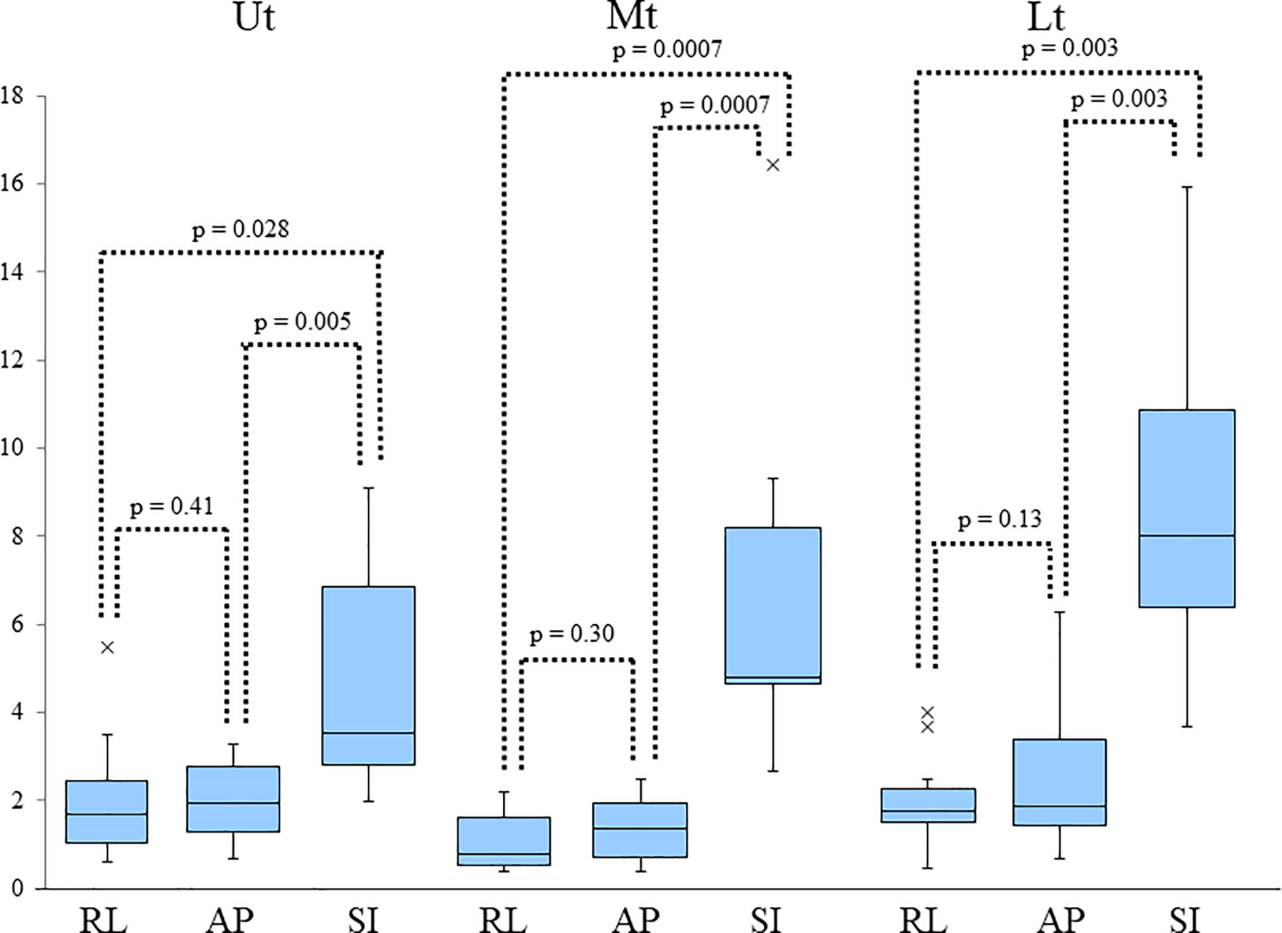
Box-and-whisker plots for the maximum absolute amplitudes of respiratory marker motion according to tumor site and motion direction. Box-and-whisker plots for the maximum absolute amplitudes of respiratory marker motion according to tumor site and motion direction. The band inside the boxes represent the median, and the bottom and top of the boxes represent the 25^th^ and 75^th^ percentiles. The whiskers indicate the lowest datum still within the 1.5 interquartile range (IQR) of the lower quartile, and the highest datum still within the 1.5 IQR of the upper quartile. Crosses (‘x’) indicate outliers of maximum displacement. The amplitudes in the SI direction were significantly larger than in all other directions in all sites. Ut: upper thoracic esophagus; Mt: middle thoracic esophagus; Lt: lower thoracic esophagus /esophagogastric junction; RL: right–left; AP: anterior–posterior; SI: superior-inferior.

Plots of respiratory marker motion in consecutive breathing phases, according to tumor site and motion direction, are given in Fig 2. The detail range of marker motion in the RL, AP, and SI directions have been listed in Table 2. Fig 3 shows box-and-whisker plots for the maximum absolute amplitudes of respiratory marker motion. The amplitudes in the SI direction were significantly higher than in the other directions, in all sites. The detail data of median absolute maximum amplitude (interquartile range: (IQR)) in the RL, AP, and SI directions have been listed in Table 2. The 95th percentile values of marker motion in RL, AP, and SI directions were 3.5 mm, 2.3 mm, and 6.5 mm, respectively, in the Ut, 1.5 mm, 2.1 mm, and 8.3 mm, respectively, in the Mt, and 3.5 mm, 4.2 mm, and 12.6 mm, respectively, in the Lt. These data summarized in Table 2.

**Table 2:** The detailed data of marker motion during free-breathing and inter-CBCT session marker displacement using breath-hold.

Location		Range	Median absolute maximum amplitudes (IQR)	The 95th percentile values of marker motion
Marker motion during free-breathing				
Ut	RL	-2.5 to 5.5	1.7(1.4)	3.5
	AP	-3.1 to 3.3	2.0 (1.5)	2.3
	SI	-9.1 to 1.1	3.6 (4.1)	6.5
Mt	RL	-1.6 to 2.2	0.8 (1.1)	1.7
	AP	-2.3 to 2.5	1.4 (1.2)	2.1
	SI	-16.4 to 2.0	4.8 (3.6)	8.3
Lt	RL	-4 to 1.8	1.8 (0.8)	3.5
	AP	-6.3 to 0.7	1.9 (2.0)	4.2
	SI	-15.9 to 0.6	8.0 (4.5)	12.6
Inter-CBCT session marker displacement using breath-hold				
Ut	RL	-2.0 to 3.3	1.3 (1.0)	1.9
	AP	-1.7 to 2.4	1.1 (0.7)	1.4
	SI	-4.1 to 5.2	3.3 (1.8)	3.9
Mt	RL	-2.6 to 1.4	0.7 (0.7)	1.5
	AP	-1.7 to 1.9	1.1 (0.4)	1.4
	SI	-4.4 to 5.6	3.4(1.4)	4.5
Lt	RL	-4.7 to 5.2	2.0 (0.8)	2.5
	AP	-3.5 to 2.1	2.6 (2.2)	3.1
	SI	-5.7 to 5.2	3.5 (1.8)	4.1

Ut: upper thoracic esophagus, Mt: middle thoracic esophagus, Lt: lower thoracic esophagus /esophagogastric junction, IQR: interquartile range, RL: right–left on the x-axis, AP: anterior–posterior on the y-axis, SI: superior-inferior on the z-axis

### Inter-CBCT session marker displacement using breath-hold

**Fig 4:**
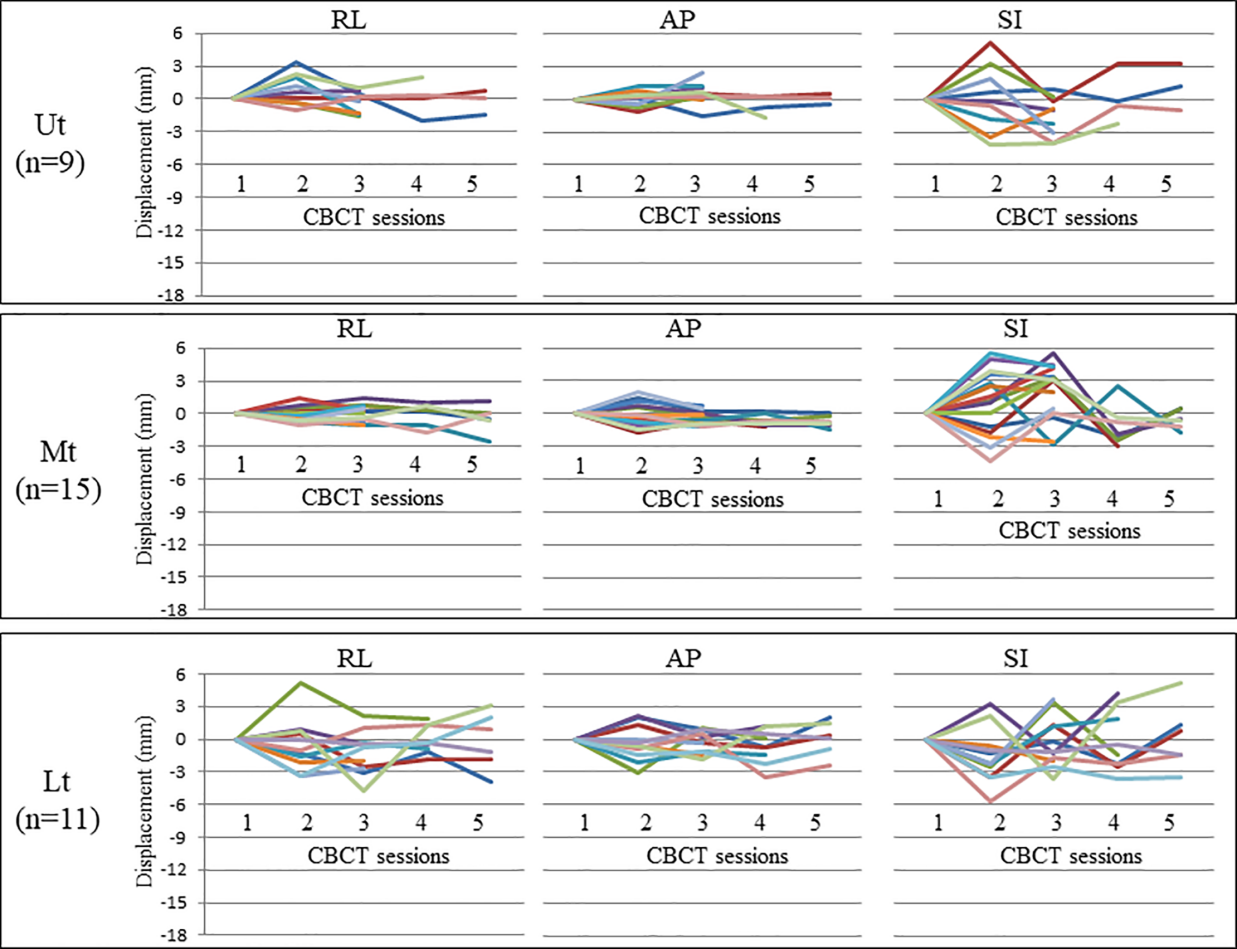
Plots of inter-CBCT session marker displacement using breath-hold. This figure provides plots of inter-CBCT session marker displacement using breath-hold. CBCT data based on the first CBCT scan are shown. Three, four, and five CBCT scans were performed in 7, 4, and 5 patients, respectively. The different colors stand for the data from the different metal markers. CBCT: Cone beam computed tomography; Ut: upper thoracic esophagus; Mt: middle thoracic esophagus; Lt: lower thoracic esophagus /esophagogastric junction; RL: right–left; AP: anterior–posterior; SI: superior-inferior.

**Fig 5:**
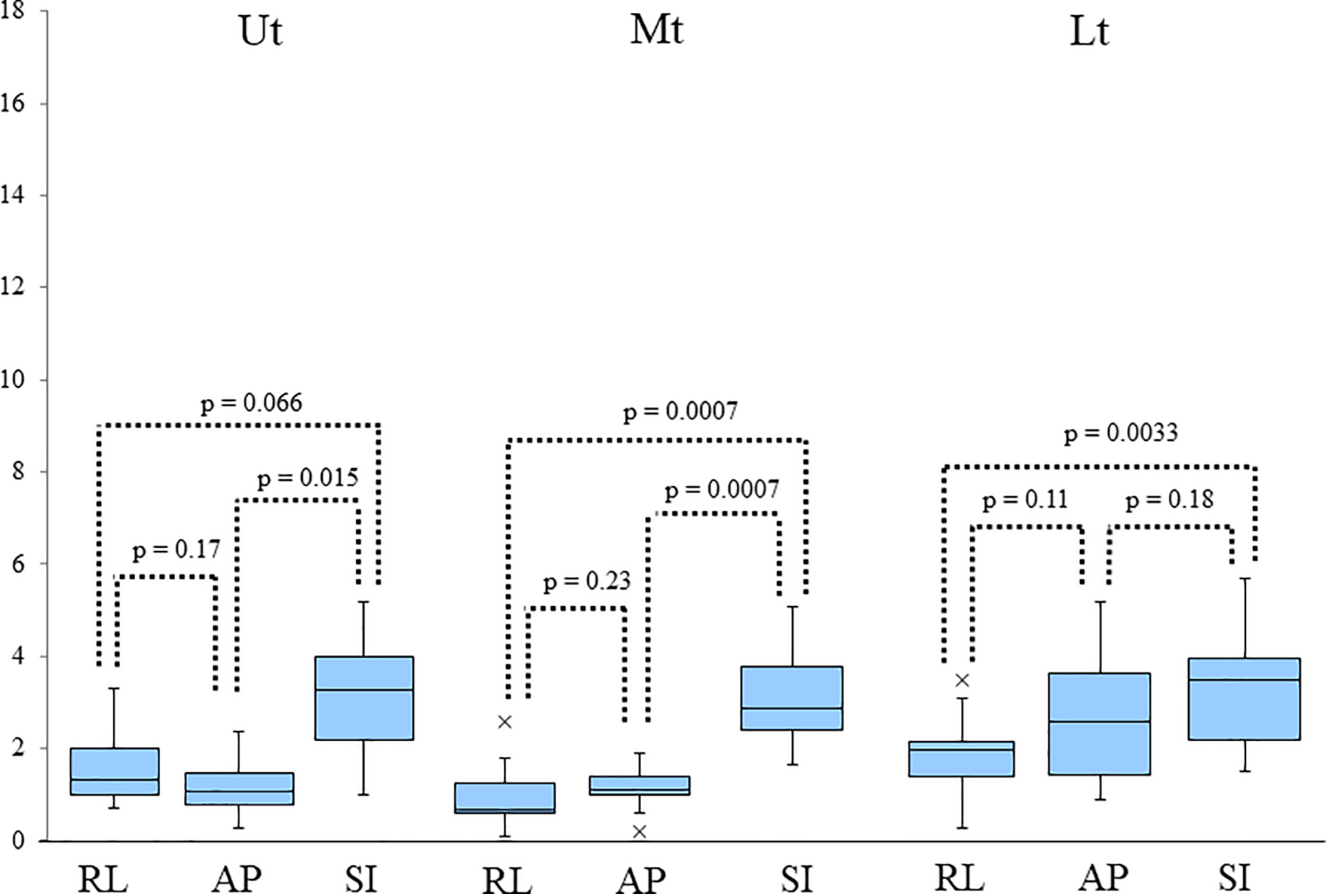
Box-and-whisker plots of the maximum absolute amplitude of inter-CBCT session marker displacement using breath-hold. Box-and-whisker plots of the maximum absolute amplitude of inter-CBCT session marker displacement using breath-hold. The amplitudes in the SI direction in all sites were significantly larger than all other directions, except the AP direction in Lt. Ut: upper thoracic esophagus; Mt: middle thoracic esophagus; Lt: lower thoracic esophagus /esophagogastric junction; RL: right–left; AP: anterior–posterior; SI: superior-inferior.

Three, four, and five CBCT scans were performed in 7, 4, and 5 patients, respectively. Fig 4 provides plots of inter-CBCT session marker displacement using breath-hold, where CBCT data was based on the first CBCT data. The detailed data of amplitude range in the RL, AP, and SI directions have been listed in Table 2. Fig 5 provides box-and-whisker plots of the maximum absolute amplitudes of inter-CBCT session marker displacement using breath-hold. The amplitudes in the SI direction in all sites were significantly higher than in all other directions, except the AP direction in the Lt. The numerical values of the median absolute maximum amplitude (IQR) in the RL, AP, and SI directions have been listed in Table 2. The 95th percentile values of marker motion in RL, AP, and SI directions were 1.9 mm, 1.4 mm, and 1.9 mm, respectively, in the Ut, 1.5 mm, 1.4 mm, and 4.5 mm, respectively, in the Mt, and 2.5 mm, 3.1 mm, and 4.1 mm, respectively, in the Lt. These data summarized in Table 2.

## Discussion

Recently, serious late cardiopulmonary toxicities after CCRT in patients with EC have been reported [5–9]. Large volumes irradiated in high doses to the heart and lungs increase the risk of cardiopulmonary toxicity, and this volume is affected by tumor size and esophageal motion. Esophageal motion consists of respiratory, heart beat-related, and peristalsis-related motions. In clinic, patients with EC usually undergo RT during free-breathing; thus, it is important to measure esophageal motion during free-breathing to determine optimal internal margins. Several methods have been reported to evaluate esophageal motion, including evaluating the motion of a manually contoured target or the esophagus itself [11–15], as well as fiducial markers placed on the esophageal wall [16,17]. In this study, we wanted to evaluate physiological esophageal motion as accurately as possible. Therefore, we selected patients with early stage (T1-2N0M0) tumors with virtually no invasion or adhesion to adjacent structures. We also used 4D-CT metal marker motion data to confirm tumor position for treatment planning (i.e. fiducial markers for esophageal motion).

Yamashita et al reported that esophageal motion, measured with fiducial markers via 4D-CT, and 95th percentiles of tumor motion in SI, AP, and RL directions, were 4.3 mm, 1.5 mm, and 2.0 mm, respectively, in the Ut, 7.4 mm, 3.0 mm, and 2.4 mm, respectively, in the Mt, and 13.8 mm, 6.6 mm, and 6.8 mm, respectively, in the Lt [16]. In our study, the 95th percentiles of tumor motion in SI, AP, and RL directions were 6.5 mm, 2.3 mm, and 3.5 mm, respectively, in the Ut, 8.3 mm, 2.1 mm, and 1.7 mm, respectively, in the Mt, and 12.6 mm, 4.2 mm, and 3.5 mm, respectively, in the Lt. Thus, results of both studies were very similar. Esophageal motion in the SI direction was larger than in other directions at all esophageal sites, and motion in the Lt was larger than in other sites. In this study, the maximum absolute motion in the SI direction was 9.1 mm in the Ut, 16.4 mm in the Mt, and 15.9 mm in the Lt. Thus, esophageal motion in the SI direction showed great variability across individuals. Moreover, esophageal motion was affected by the degree of tumor invasion or adhesion to adjacent organs, and the presence of respiration according to the patient’s general condition [18]. In RT for EC, uniform internal margins should be avoided; we recommend determining an appropriate margin after investigating individual esophageal motion, prior to treatment planning.

Due to advances in RT techniques, irradiation during breath-control is now possible. There are various irradiation methods during controlled respiratory tumor motion, including the breath-hold, respiratory-gated, and the real-time tumor tracking methods, which are commonly used in the treatment of lung cancer. Breath-hold technique is now particularly widely used in stereotactic body radiation therapy for small lung tumors, due to its good reproducibility of the target position [19]. We propose that the breath-hold technique should be adopted for EC patients, given that esophageal motion is comparatively large and varies greatly between individuals. Additionally, to decrease the risk of cardiac toxicity after definitive RT for EC, utilization of intensity modulated radiation therapy (IMRT) is recommended. Lin et al reported that a decrease in cardiac dose by IMRT led to significant improvements in cardiac-related mortality [20, 21]. However, in IMRT for EC, esophageal positions during free breathing might be negatively affected. Wang et al reported inter-fractional displacement of the GEJ during IMRT in patients with EC [11]. They found marked inter-fractional displacements in the GEJ in the SI direction (absolute mean, 6.77 mm; maximum displacement, 17.6 mm). Additionally, they showed that systematic displacement in an inferior direction resulted in higher-than-intended doses to the GEJ, with increased hot-spots to the adjacent stomach and lung base. They concluded that improved allowance for depth of breathing is required to reduce inter-fractional variability. In this study, we examined inter-CBCT session marker displacement using breath-hold. There have been several reports of inter-fractional esophageal displacement during free-breathing [22–24]. However, to our knowledge, there have been no reports of reproducibility of the esophageal position using breath-hold in patients with EC. In our study, the inter-CBCT session marker displacement using breath-hold coverage in 95% values, in SI, AP, and RL directions, were 3.9 mm, 1.4 mm, and 1.9 mm, respectively, in the Ut, 4.5 mm, 1.4 mm, and 1.5 mm in the Mt, and 4.1 mm, 3.1 mm, and 2.5 mm in the Lt. The esophageal motion amplitudes during breath-hold were smaller than during free-breathing. Moreover, compared with the data reported by Wang et al [11], the values for inter-CBCT session marker displacement using breath-hold in the Lt were much smaller. Individual displacement differences were also small. Thus, applying the breath-hold method for IMRT in patients with EC may be effective for preventing toxicities of organs at risk. As the new method of CBCT, Kincaid Jr et al. reported the efficacy of respiratory motion-corrected-CBCT in improved organ visibility and localization accuracy for gated treatment at end expiration in the GEJ cancer cases [25].

There are several studies, which have evaluated the visibility and artifacts created by various materials used as fiducial markers such as polymer, carbon and gold, for image-guided radiotherapy (IGRT) [26, 27]. Handsfield et al. reported that the verification and selection of an optimal fiducial marker depends on the imaging modality for IGRT [26]. Chan et al. examined the suitable combination of materials and the diameters of commercially available fiducial markers [27]. Liu et al. showed that fiducial markers were useful surrogates when using respiratory gating to reduce motion of GEJ tumors [28]. In our study, we used metallic clips, which were placed on the wall of the esophagus, as markers for the measurement of esophageal movement. Visibility was enhanced for all metallic markers with few artifacts in the CBCT images. When IGRT is performed with breath-hold, as in the case of esophageal cancer, it is necessary to select an implantable fiducial marker using the optimal material according to the imaging modality.

Our study was limited by the small number of patients. Moreover, the acquired CBCT data was obtained based on voluntary deep expiration breath-holds for 3–4 times, without using a respiratory monitoring device. Therefore, our data might under-or over-estimate the esophageal motion amplitude. Sheng et al. evaluated the intrafractional seminal vesicle (SV) motion relative to the prostate using CBCT images and suggested a benchmark for SV margins [29]. We will attempt to perform more accurate data acquisition and analysis in a future study, with the goal of determining the optimal margin for the esophagus under breath-hold, taking into consideration intrafractional esophageal motion. Based on this basic research towards the introduction of IMRT during breath-hold for thoracic patients with EC, we aim to develop an optimal IMRT planning system to decrease the risk of late cardiac toxicity.

## Conclusion

During free-breathing, esophageal motion in the SI direction is larger in all esophagus sites than in the other directions. Amplitudes vary substantially between individuals. The breath-hold technique is feasible for minimizing esophageal displacement during RT in patients with EC.
